# Combined association of cognitive impairment and poor oral health on mortality risk in older adults: Results from the NHANES with 15 years of follow‐up

**DOI:** 10.1002/JPER.21-0292

**Published:** 2021-11-12

**Authors:** An Li, Yuntao Chen, Anita Visser, Luc A.M. Marks, Geerten‐Has E. Tjakkes

**Affiliations:** ^1^ Center for Dentistry and Oral Hygiene University Medical Center Groningen, University of Groningen Groningen the Netherlands; ^2^ Stomatological Hospital Southern Medical University Guangzhou China; ^3^ Medical Statistics and Decision‐Making, Department of Epidemiology University Medical Center Groningen, University of Groningen Groningen the Netherlands; ^4^ Department of Maxillofacial Surgery University Medical Center Groningen, University of Groningen Groningen the Netherlands

**Keywords:** caries, cognitive impairment, edentulous, mortality, periodontitis

## Abstract

**Background:**

Cognitive impairment and poor oral health are frequently seen among older adults. Both conditions have been identified as risk factors for mortality. However, the combined associations of cognitive impairment and poor oral health with mortality have not been well studied and are therefore the aim of this cohort study.

**Methods:**

We analyzed data from the National Health and Nutrition Examination Survey (1999–2002) linked with mortality data obtained from the 2015 public‐use linked mortality file. Cognitive impairment was defined as a digit symbol substitution test score lower than the lowest quartile. Oral health status was assessed based on presence of untreated caries, moderate to severe periodontitis, and edentulism. The combined effects of caries/periodontitis or edentulism and cognitive impairment on all‐cause and cardiometabolic mortality were examined using the Cox proportional hazard models after adjusting for potential confounders including demographic characteristics, lifestyle, biomarkers, and comorbidities.

**Results:**

In total, 1973 participants were enrolled in the prospective study. At a median follow‐up of 13.4 years, 978 participants had died (264 deaths because of cardiometabolic disease). Cognitive impairment, periodontitis, and edentulism were each found to be significant predictors of all‐cause mortality. Caries, however, was not significantly related to mortality. When analyzing these predictors in combination, a diagnosis of cognitive impairment and periodontitis was associated with an 83.1% increase in all‐cause mortality risk and an 87.7% increase in cardiometabolic mortality risk compared with healthy controls. Similarly, the risk for all‐cause mortality was highest in cases where impaired cognition and edentulism co‐occurred (adjusted hazard ratio = 1.701, 1.338–2.161).

**Conclusion:**

Concomitant presence of cognitive impairment and periodontitis or edentulism can be associated with a higher risk of mortality among older U.S. adults.

## 1 INTRODUCTION

Longer life expectancy has been increasing the proportion of older persons in industrialized countries^1^ and led to a commensurate increase in the prevalence of Alzheimer disease (AD).^2^ Cognitive impairment is a common phenomenon in older adults and can be seen as a prodrome of AD.^3^ Even at mild levels, cognitive impairment has emerged as a major contributing factor to mortality.^4^, ^5^, ^6^ Also, cognitive impairments are considered the main barriers to maintaining routine dental visits and good oral hygiene care in older adults.^7^, ^8^, ^9^ Oral health problems (e.g., periodontal inflammation, dental caries, and masticatory dysfunction) are highly prevalent among older adults, giving rise to other health problems^10^ and being a risk indicator of mortality.^11^

Vascular risk factors and diseases might explain the increased mortality associated with dementia or oral diseases to some extent. Older people with cognitive impairment and systemic vascular comorbidities (e.g., high blood pressure and diabetes mellitus) may have increased mortality risk.^12^, ^13^ In a recent systematic review, poor oral health was found to be associated with mortality.^14^ A considerable proportion of such mortality could be attributed to comorbid conditions.^15^ Therefore, it is plausible that cognitive impairment or oral diseases may separately increase the risk of cardiometabolic death.

Emerging evidence indicates that cognitive impairment and poor oral health are closely intertwined,^16^, ^17^, ^18^ and their relationship could be bidirectional.^19^, ^20^ As a cost‐effective method, maintaining oral health conditions seems crucial in reducing complications and deaths, especially in the older populations with cognitive decline. However, studies on the interrelationships between cognitive function, oral health, and mortality are scarce. Hence, we aim to investigate the potential joint effects of cognitive impairment and poor oral health (periodontitis, caries, or edentulism) on all‐cause and cardiometabolic mortality among older Americans in a cohort study with a follow‐up duration of 15 years.

## 2 METHODS

### 2.1 Study design and analytical sample

Data from older adults aged 60 years and older who participated in the U.S. National Health and Nutrition Examination Survey (NHANES, 1999–2000^21^ and 2001–2002^22^) were linked to public‐use mortality files for the period up to December 2015. The NHANES is a stratified, multistage, clustered probability sampling study focused on U.S. civilians' health and nutritional status; it is administered by the U.S. National Center for Health Statistics (NCHS). The survey consists of an in‐home interview followed by physical examinations and biological sample collections at a mobile examination center. The NCHS Ethics Review Board approved all NHANES protocols and testing procedures (including a series of questionnaires, physical, and biochemical exams, cognitive function assessment, and a comprehensive dental exam), and all participants provided written informed consent.^23^ The prospective cohort study conforms to the Strengthening the Reporting of Observational Studies in Epidemiology (STROBE) guideline^24^ (see Supplemental Figure S1 in online *Journal of Periodontology*).

### 2.2 Covariates

Baseline sociodemographic, behavioral, and clinical variables were used as covariates. Sociodemographic variables included age, gender, race/ethnicity, educational level, and annual household income. Race/ethnicity was categorized into non‐Hispanic white, non‐Hispanic black, and others. Educational level was grouped into high school/less, some college, and higher. Annual household income was grouped into <$20,000, $20,000–$75,000, and >$75,000. Behavioral variables included smoking status (never, former, and current smoker), alcohol intake (<12 and ≥12 drinks/year), and the time since last dental visit (<1, 1–3, > 3 years). We used dietary data obtained from 24‐hour recall to calculate the Healthy Eating Index‐2015 (HEI‐2015) for assessing the dietary quality of the NHANES study population.^25^, ^26^ The HEI‐2015 contains 13 components that sum to a maximum score of 100 points (see Supplemental Table S1 in online *Journal of Periodontology*). For details, see Supplemental Methods. A higher score means better adherence to the Dietary Guidelines for Americans

Clinical variables included cardiovascular disease risk factors (CVD‐RF) and medical conditions. CVD‐RF included obesity, hypertension, dyslipidemia, and diabetes mellitus, as described in previous study.^27^ We calculated the total number of CVD‐RF for each individual (range 0–4). The detailed definitions and categories of these risk factors (i.e., obesity, hypertension, dyslipidemia, and diabetes mellitus) are presented in Supplemental Table S2 in the online *Journal of Periodontology*. Medical condition included elevated systemic inflammation (as measured by C‐reactive protein [CRP]) level, heart disease, and stroke. Table S2 also presents the definitions for these medical conditions.

### 2.3 Assessment of cognitive function

The digit symbol substitution test (DSST) was administered to assess cognitive function.^28^ Participants were asked to draw as many symbols paired with numbers as possible within 2 minutes. Following the standard scoring method, a score was given for each correctly drawn symbol (with the maximum score being 133). A higher score represents better cognitive function. Because there are no standard norms for the DSST, we defined scores falling below the lowest quartile (≤29 scores) in the study group as indicating cognitive impairment, consistent with the methods previously described^29^, ^30^; the higher quartiles were considered as the normal cognition.

### 2.4 Oral health outcomes

Oral health outcomes were assessed based on periodontal status, dental caries, and edentulism. Trained and calibrated dentists from the NCHS examined each participant's oral health status. The NHANES 1999–2002 used National Institute for Dental Research periodontal probes and the random half‐mouth protocol (RHMP). One maxillary quadrant and one mandibular quadrant were randomly chosen, and periodontal status was assessed by probing assessments for clinical attachment loss (CAL) and probing pocket depth (PPD). In 1999–2000, two probing sites per tooth (mesial buccal and mid buccal) were measured, whereas, for 2001–2002, these assessments were additionally conducted at distal buccal sites. In addition, bleeding on probing was measured at three sites per tooth only in the NHANES 2001–2002. In the present study, mean CAL and mean PPD were calculated using two probing sites for consistency with the NHANES 1999–2000.^31^ As the third molars were excluded, a maximum of 14 teeth and 28 sites per individual could be examined to assess periodontal status.

We used the half‐reduced CDC/AAP (Centers for Disease Control & Prevention and American Academy of Periodontology) case definition to define periodontal disease.^32^ No/mild periodontitis was defined as no evidence of moderate or severe periodontitis; Moderate: ≥1 inter‐proximal site with ≥4 mm CAL, or ≥1 interproximal site with ≥5 mm PPD; Severe: ≥1 interproximal site with ≥6 mm CAL and ≥1 interproximal site with ≥5 mm PPD. The participants were dichotomized as no/mild periodontitis and moderate/severe periodontitis.

Regarding dental caries, the presence of decayed permanent tooth surfaces in an individual was defined as untreated caries; no decay in any tooth surface was set as the reference. Participants with no natural teeth (tooth positions presenting edentulous) were defined as the edentulous population; the others were set as the dentulous population.

### 2.5 Mortality

The primary outcome was mortality from all causes. The secondary outcome was cardiometabolic (CM) mortality. To increase the statistical power, we combined mortality because of heart disease, cerebrovascular disease, or diabetes mellitus into a single outcome (i.e., CM mortality), as previously reported.^33^ Mortality status and cause of death were determined based on publicly accessible mortality files.^34^ The follow‐up started from the interview date and ended at death or at the end of the study (December 31, 2015), whichever came first, as described before.^35^

### 2.6 Statistical analyses

Descriptive statistics were calculated to describe the characteristics of participants. Continuous variables were reported as mean with standard deviation or median with interquartile range. Categorical variables were described as numbers with frequencies. Characteristics of participants were stratified by cognitive function status and oral health outcomes and compared using one‐way ANOVA and Chi‐square.

**FIGURE 1:**
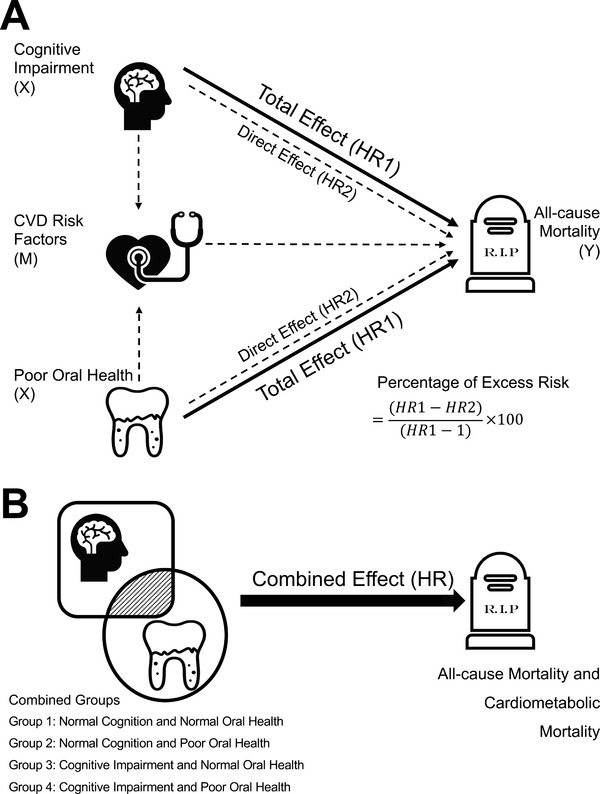
Schematic diagrams illustrating the statistical analysis. (**A**) Mediation models of the separate effects of cognitive impairment or oral disease on all‐cause mortality, using cardiometabolic risk as a mediator. (**B**) The combined effects of cognitive impairment and oral disease on all‐cause mortality and cardiometabolic mortality

We studied the interrelationship between cognitive impairment, poor oral health, and mortality in two steps. First, we estimated the extent to which the effects of cognitive impairment or oral disease (exposure variables) on all‐cause mortality (outcome variable) are attributable to CVD‐RF (potential mediator) (see Figure 1A). Multinominal log‐linear models were conducted to assess the associations between exposure variables and the potential mediator. Then, Cox proportional hazards models were used to estimate hazard ratios (HRs) and 95% confidence intervals (CIs) for all‐cause mortality. In the crude model, estimates were not adjusted. Model 1 was adjusted for sociodemographic variables (age, gender, race/ethnicity, education level, and income level) behavioral variables (smoking, drinking, and dental visit), and medical conditions (elevated systemic inflammation, heart disease, and stroke). Model 2 was further adjusted for CVD‐RF. The formula $\left(HR_{1}-HR_{2}\right)/\left(HR_{1}-1\right)\times 100$ was used to determine the percentage of excess risk (hazard) explained by CVD‐RF,^36^ where $HR_{1}$ is the HR of Model 1 and $HR_{2}$ is the HR of Model 2. Given no interaction between exposure and mediator on the outcome, the estimation of mediated proportion is based on comparing the total effect and the controlled direct effect;^37^ Figure 1A).

Second, four mutually exclusive risk groups were created for the joint effect of cognitive performance and oral health status (Figure 1B): Group 1, normal cognition and normal oral health; Group 2, normal cognition and poor oral health; Group 3, cognitive impairment and normal oral health; and Group 4, cognitive impairment and poor oral health. The cumulative mortality curves of the four combined groups were computed using cumulative incidence function. The Cox model was used to estimate the combined associations of cognitive impairment and oral disease with all‐cause mortality, and a cause‐specific Cox model was used for CM mortality (Figure 1B). All models were adjusted for the aforementioned sociodemographic variables, behavioral variables, medical conditions, and CVD‐RF. Next, interaction analyses were performed to explore the potential interaction between cognitive impairment and oral disease.

Furthermore, we conducted several sensitivity analyses to demonstrate the robustness of the results. Firstly, we defined periodontal health according to the joint workshop of the EFP/AAP (European Federation of Periodontology & American Academy of Periodontology)^38^ and repeated the Cox regression analyses of combined effects in the NHANES 2001–2002. Periodontal health was defined as no sites with PPD > 3 mm and < 10% of sites with bleeding on probing in an intact periodontium (CAL < 3 mm); the others were set as a periodontal disease. Secondly, we also repeated the Cox regression analyses in the NHANES 1999–2002 when individuals having eight teeth or fewer were used to define severe tooth loss.^39^ Considering the low percentage of the missing data from the covariates analyzed, we conducted complete‐case multivariable regression. All data analyses were performed with the R Project for Statistical Computing (version 3.6.0) and SPSS 25 (SPSS Inc., Chicago, IL, USA). A *P*‐value of less than 0.05 was considered statistically significant.

## 3 RESULTS

### 3.1 Characteristics of the study population at baseline

Of the 3706 older American in the NHANES dataset who were eligible to participate in this study, 2975 adults completed the cognitive assessment. Older participants who did not have data on dental examination (*n* = 1002) were excluded, resulting in an analytical sample of 1973 participants. Among those participants, a total of 1478 older persons were regarded as the dentate subpopulation having periodontal and caries data, as depicted in Supplemental Figure S2 in the online *Journal of Periodontology*. The mean age was 70.68 ± 7.70 years; of the population, 50% were men and 58.2% non‐Hispanic (Table **1**). By the median follow‐up of 13.4 years, 978 participants had died. Among those, 264 deaths (27%) were because of CM disease. Mean DSST score was 41.69 (range: 0–117). In addition, baseline statistics stratified by cognitive function status and oral health outcomes are displayed in Supplemental Table S3 in the online *Journal of Periodontology*.

**TABLE 1:** Characteristics of study participants in NHANES 1999–2002 (*n* = 1973)

VARIABLES	NHANES 1999–2002
**Sociodemographic variables**	
Age (year), mean (SD)	70.68 (7.70)
Male, *n* (%)	987 (50.0)
Race/ethnicity, *n* (%)	
Non‐Hispanic white	1148 (58.2)
Non‐Hispanic black	307 (15.6)
Other race, including multiracial	518 (26.3)
Education level, *n* (%)^1^	
≤ High school	1287 (65.3)
College	390 (19.8)
> College	293 (14.9)
Annual household income, *n* (%)^1^	
< 20,000$	745 (39.3)
20,000–75,000$	971 (51.2)
> 75,000$	179 (9.4)
**Lifestyle variables**	
Smoking habit, *n* (%)^1^	
Non smoker	909 (46.2)
Former smoker	805 (40.9)
Current smoker	255 (13.0)
Alcohol intake > 12 drinks/year, *n* (%)^1^	1183 (61.2)
Time since the last dental visit, *n* (%)^1^	
Less than 1 year	1040 (52.8)
1‐3 years	320 (16.3)
More than 3 years	608 (30.9)
Healthy eating index‐2015, mean (SD)	54.13 (11.57)
**Cardiovascular disease risk factors**	
Body mass index (kg/m^2^), mean (SD)	28.19 (5.28)
Obesity, *n* (%)^1^	600 (31.2)
SBP (mmHg), mean (SD)	139.68 (21.88)
DBP (mmHg), mean (SD)	69.73 (15.13)
Hypertension, *n* (%)^1^	942 (48.0)
Non‐HDL cholesterol (mg/dL), mean (SD)	160.14 (39.77)
Dyslipidemia, *n* (%)^1^	945 (49.5)
Glycohemoglobin (%), mean (SD)	5.90 (1.16)
Diabetes mellitus, *n* (%)^1^	328 (17.0)
CVD risk factors, *n* (%)^1^ ^,^ ^2^	
0	308 (15.6)
1	782 (39.6)
2	630 (31.9)
3	221 (11.2)
4	32 (1.6)
**Medical conditions**	
C‐reactive protein^3^	0.28 (0.43)
Elevated systemic inflammation, *n* (%)^1^	636 (33.8)
Heart disease, *n* (%)^1^	306 (15.5)
Stroke, *n* (%)^1^	115 (5.8)
**Exposure variables** ^4^	
Caries status, *n* (%)^5^	
Untreated caries	1150 (58.3)
No untreated caries	328 (16.6)
Periodontal status, *n* (%)^5^	
No/mild periodontitis	746 (37.8)
Moderate/severe periodontitis	732 (37.1)
Dentate status, *n* (%)	
Dentulous population	1478 (74.9)
Edentulous population	495 (25.1)
Cognitive status, *n* (%)	
Normal cognition	1464 (74.2)
Cognitive impairment	509 (25.8)
**Outcome variables** ^6^	
Mortality status, *n* (%)	
All‐cause mortality	978 (49.6)
Cardiometabolic mortality	264 (13.4)

Abbreviations: AAP, American Academy of Periodontology; CDC, Centers for Disease Control and Prevention; CRP, C‐reactive protein; CVD, cardiovascular disease; DBP, diastolic blood pressure; DSST, digit symbol substitution test; HDL, high‐density lipoprotein; SBP, systolic blood pressure; SD, standard deviation.

^1^ Missing value for total study: education (*n* = 3; < 1%), income (*n* = 78; 4.0%), smoking (*n* = 4; < 1%), alcohol (*n *= 40; 2.0%), dental visit (*n *= 5; < 1%), hypertension (*n* = 3; < 1%), diabetes (*n* = 30; 3%), obesity (*n* = 52; 2.6%), CVD‐RF (*n* = 164; 8.3%), elevated systemic inflammation (*n* = 90; 4.6%), dyslipidemia (*n* = 63; 3.2%), heart disease (*n* = 15; < 1%), and stroke (*n* = 6; < 1%).

^2^ The risk factors of cardiovascular disease include obesity, hypertension, dyslipidemia, or diabetes mellitus^27^.

^3^ Non‐normal distribution continuous variable, median (interquartile range).

^4^ Cognitive impairment defined as DSST score ≤ 29 (the lowest quartile of score in population); Moderate/severe periodontitis was defined by the CDC/AAP case definition; Edentulism was defined as the complete loss of all‐natural teeth; Untreated caries was defined as having any decayed permanent tooth surfaces.

^5^ There was no caries and periodontal data in the subgroup of edentulous population (*n* = 495; 25.1%).

^6^ The median follow‐up period for all‐cause mortality was 13.4 years.

### 3.2 Separate associations with all‐cause mortality

When considered separately, cognitive impairment, periodontal disease, and edentulism were all significantly associated with risk of CVD‐RF (see Supplemental Table S4 in online *Journal of Periodontology*). However, untreated caries was not statistically associated with CVD‐RF (Table S4). A similar pattern emerged for all‐cause mortality. Cognitive impairment, moderate/severe periodontitis, or edentulism was significantly associated with all‐cause mortality in unadjusted and adjusted models. Specifically, the HR for mortality with cognitive impairment was 1.461 (Model 1) before and 1.385 (Model 2) after adjusting for CVD‐RF, a reduction of 16.5% (Table 2). Regarding oral disease, CVD‐RF also attenuated the HR of periodontitis for all‐cause mortality by 18.5%. Similarly, the estimates of HR were reduced by 16.1% in the edentulous population (Table 2).

**TABLE 2:** The separate associations of cognitive impairment or caries/periodontitis/edentulism with all‐cause mortality

All‐Cause Mortality	Cases/Participants	Crude Model HR _unadjusted_ (95% CI)	Model 1^1^ HR1 (95% CI)	Model 2^1^ HR2 (95% CI)	% Excess risk explained^2^
Cognitive Performance					
Normal cognition	647/1464	1 [Reference]	1 [Reference]	1 [Reference]	16.5%
Cognitive impairment	331/509	**1.760 (1.511 to 2.050)**	**1.461 (1.235 to 1.728)**	**1.385 (1.170 to 1.640)**	
Caries Status					
No untreated caries	456/1150	1 [Reference]	1 [Reference]	1 [Reference]	5.7%
Untreated caries	165/328	**1.366 (1.120 to 1.665)**	1.174 (0.951 to 1.450)	1.164 (0.943 to 1.438)	
Periodontal Status					
No/mild periodontitis	249/746	1 [Reference]	1 [Reference]	1 [Reference]	18.5%
Moderate/severe periodontitis	372/732	**1.758 (1.471 to 2.102)**	**1.303 (1.094 to 1.552)**	**1.247 (1.044 to 1.489)**	
Dentate Status					
Dentulous population	621/1478	1 [Reference]	1 [Reference]	1 [Reference]	16.1%
Edentulous population	357/495	**2.213 (1.906 to 2.570)**	**1.361 (1.152 to 1.607)**	**1.303 (1.103 to 1.540)**	

Abbreviations: CI, confidence interval; CVD‐RF, cardiovascular disease risk factors; HR, hazard ratio.

^1^Model 1 was adjusted for sociodemographic variables (age, gender, race/ethnicity, educational level, and income level), behavioral variables (smoking, drinking, and dental visit), and medical conditions (elevated systemic inflammation, heart disease, and stroke). Model 2 was further adjusted for CVD‐RF. The risk factors of cardiovascular disease include obesity, hypertension, dyslipidemia, or diabetes mellitus.^27^

^2^ The percentages of excess risk (hazard) explained by CVD‐RF were calculated using the formula $\left(HR_{1}-HR_{2}\right)/\left(HR_{1}-1\right)*100.$

Bold indicates *P*‐value < 0.05.

### 3.3 Combined effects on all‐cause and cardiometabolic mortality

**FIGURE 2:**
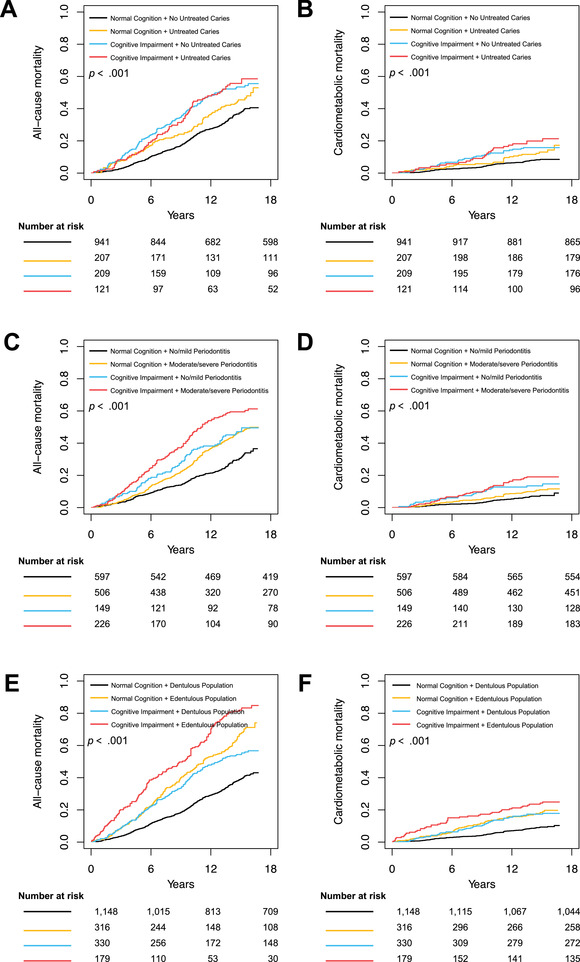
Cumulative mortality rates among the four combined groups. All‐cause mortality for cognitive impairment combined with untreated caries (**A**), periodontal disease (**C**), or edentulism (**E**). Cardiometabolic mortality for cognitive impairment combined untreated caries (**B**), periodontal disease (**D**), or edentulism (**F**). *P* value denotes the difference between the survival curves

The results of separate associations showed that CVD‐RF partly explained the risk of total mortality. Therefore, we considered CM mortality as the secondary outcome in a biologically plausible way. We found only cognitive impairment or periodontitis was associated with CM mortality separately (see Supplemental Table S5 in online *Journal of Periodontology*). Figure 2 shows the cumulative mortality curves for the four combined groups. When analyzing the combined effects, participants with cognitive impairment and periodontitis or edentulism (Group 4) tended to have the highest all‐cause (Figure 2C, E) and CM mortality (Figure 2D, F). These trends are less marked in the combined group of untreated caries and cognitive impairment (Figure 2A, B).

As shown in Table 3, participants with cognitive impairment and periodontitis had higher all‐cause (HR _adjusted_ = 1.831, 95% CI: 1.377–2.434) and cardiometabolic (HR _adjusted_ = 1.877, 95% CI: 1.102–3.197) mortality than the healthy controls (Group 1). The edentulous population with cognitive impairment was 70.1% more likely to suffer all‐cause mortality but was not significantly associated with increased CM mortality (Table 3) compared with those in Group 1. However, when cognition and caries were combined, the progressive and additive trends on mortality were not obvious (Table 3). Furthermore, we did not detect any statistically significant interaction effects of cognitive impairment and caries/periodontitis/ edentulism on all‐cause and CM mortality. Sensitivity analyses were consistent with the primary analysis (see Supplemental Table S6 and Table S7 in online *Journal of Periodontology*).

**TABLE 3:** The combined associations of cognitive impairment and caries/periodontitis/ edentulism with all‐cause and cardiometabolic mortality

	All‐Cause Mortality			Cardiometabolic Mortality^4^		
	Cases/Participants	Adjusted HR^1^ (95% CI)	*P* for Interaction^3^	Cases/Participants	Adjusted HR^1^ (95% CI)	*P* for Interaction^3^
Cognitive performance combined with caries status^2^						
Group 1	343/941	1 [Reference]	.444	76/941	1 [Reference]	0.554
Group 2	96/207	1.223 (0.945–1.583)		28/207	1.508 (0.935–2.432)	
Group 3	113/209	**1.526 (1.173**–**1.984)**		33/209	1.711 (0.971‐2.878)	
Group 4	69/121	**1.589 (1.142**–**2.211)**		25/121	1.878 (0.989–3.203)	
Cognitive performance combined with periodontal status^2^						
Group 1	178/597	1 [Reference]	.079	43/597	1 [Reference]	.203
Group 2	236/506	**1.309 (1.061**–**1.615)**		55/506	1.269 (0.828–1.947)	
Group 3	71/149	**1.703 (1.234**–**2.351)**		21/149	1.735 (0.991‐3.361)	
Group 4	136/226	**1.831 (1.377**–**2.434)**		43/226	**1.877 (1.102**‐**3.197)**	
Cognitive performance combined with dentate status^2^						
Group 1	439/1148	1 [Reference]	.245	104/1148	1 [Reference]	.329
Group 2	208/316	**1.377 (1.121**–**1.691)**		58/316	1.349 (0.912–1.996)	
Group 3	182/330	**1.479 (1.193**–**1.832)**		58/330	1.505 (0.906–2.271)	
Group 4	149/179	**1.701 (1.338**–**2.161)**		44/179	1.638 (0.947–2.564)	

Abbreviations: HR, hazard ratio; CI, confidence interval.

^1^ Multivariable Cox proportional hazards models were adjusted for sociodemographic variables, behavioral variables, medical conditions, and cardiovascular disease risk factors.

^2^ Four combined groups see the legend of Figure 1.

^3^ In the interaction analyses, we included an interaction term (cognitive status * caries/periodontitis/ edentulism).

^4^ Cardiometabolic mortality combined diseases of heart, cerebrovascular diseases, and diabetes mellitus.

Bold indicates *P*‐value < 0.05.

## 4 DISCUSSION

In this prospective cohort study, the association of cognitive impairment and poor oral health with mortality in older people was investigated. Cognitive impairment, periodontitis, and edentulism were associated with increased all‐cause mortality, whereas untreated caries was not related to mortality. Cardiovascular disease risk factors may play an explanatory role in the association of each significant predictor with all‐cause mortality. All‐cause and cardiometabolic mortality risk significantly increased among older people with coexisting periodontitis and impaired cognition when compared to healthy controls. Similarly, the risk of all‐cause death was highest in the participants with coexisting edentulism and impaired cognition.

The strengths of this study include the considerable follow‐up duration and event rate. The findings persisted after adjusting for a range of potential confounders and were robust in the sensitivity analysis. Our findings indicated the combined effect of impaired cognition and poor oral health on mortality, an association that has rarely been studied previously. Cognitive impairment and edentulism were found to increase the risk of mortality in a Spanish geriatric population from nursing homes.^40^ Marín‐Zuluaga et al. demonstrated an association of cognition–oral health with mortality and supports the findings of the current study, although with a different source population.

Plausible biological mechanisms for an increased mortality risk may involve the shared CVD‐RF. The separate association of impaired cognition, periodontitis, and edentulism with all‐cause mortality was attenuated but still statistically significant in the fully adjusted model. The finding suggests that CVD‐RF may partly explain the excess risk of total death, which is consistent with prior studies.^14^, ^41^ Correspondingly, periodontitis combined with cognitive impairment increased cardiometabolic mortality. Periodontal pathogens may increase incident dementia and potentially the risk of AD‐associated mortality through a chronic low‐grade inflammatory response, oral microbial dysbiosis, and bacterial invasion.^42^, ^43^, ^44^ Edentulism affects masticatory function and nutrition intake and is concomitant with many systemic diseases.^45^, ^46^ The long‐term cumulative effects of oral infection or edentulism may contribute to neurological dysfunction^47^ and affect a spectrum of cardiometabolic complications,^48^ thus ultimately increasing death risk in older persons.

In terms of clinical implications, the present study's findings raise awareness of the importance of interdisciplinary efforts between geriatric physicians and dentists. Most phase III clinical trials in AD therapeutics have not been found to change the disease's trajectory,^49^, ^50^, ^51^, ^52^ indicating that no well‐studied pharmacotherapy is available. The emphasis has increasingly shifted from pharmacotherapy to prevention. Delaying the onset and/or progression of AD by only 1 year would result in a reduction of 9.2 million cases by 2050.^2^ Notably, dental prophylaxis and periodontal treatment had beneficial effects in reducing the occurrence and progression of dementia in a large‐scale cohort study.^53^ However, physicians often neglect the implications of poor oral health in the vulnerable elderly.

This study highlights the key combinations of oral diseases and cognitive impairments, demonstrating poor periodontal health increases CVD risk, also associated with cognitive function. In turn, the comorbidities may increase the mortality risk synergistically. Great care should be taken to prevent periodontitis and loss of all teeth (edentulism), as this increases the risk for mortality significantly, particularly in the elderly with cognitive impairment.^54^ From a health economics perspective, providing oral health advice, and maintaining periodontal health seems a more cost‐effective strategy when compared to intervening in other systemic diseases and lifestyles.^54^ Prevention and management of periodontitis and tooth loss are expected to prevent the onset and development of cognitive impairment and improve the quality and span of life. To summarize, maintaining oral health care in older people with cognitive impairment could be a crux of reducing the mortality risk.

Several potential limitations may had affected the interpretation of the findings. First, the RHMP may have underestimated the prevalence and severity of periodontal disease compared to full‐mouth examination of six sites on all teeth.^55^ The potential bias, however, can be significantly lessened by using the half‐reduced CDC/AAP case definition^32^ that was used in the present study. In addition, cognitive function cannot be comprehensively evaluated using a single instrument in the present study, the DSST test. Although the DSST is more sensitive to executive function/processing speed than many other measures,^56^ several other cognitive deficits (e.g., memory, attention, language) should be assessed in future research. In addition, cognitive performance, oral health, and CVD‐RF were assessed only at baseline, with no subsequent changes being examined at follow‐up period. These changes may potentially affect the relationship between cognitive performance or oral health status and survival over time.

## 5 CONCLUSION

In summary, cognitive impairment, periodontitis, and edentulism seem to be significant predictors of all‐cause mortality in the U.S. geriatric population, with CVD‐RF partially explaining these associations. The combination of poor cognitive performance and periodontitis or edentulism was found to increase mortality in a progressive and additive manner. Special care should be taken in maintaining a healthy oral environment and functional mouth to prolong life span in older adults, especially for those with concomitant cognitive impairment.

## CONFLICTS OF INTEREST

The authors declare that they have no known competing financial interests or personal relationships that may have influenced the work reported in this paper. Ethical approval was not required.

## AUTHOR CONTRIBUTIONS

An Li was responsible for study conception and design, data analysis and interpretation, and the drafted manuscript; Yuntao Chen, a statistical consultant, contributed to the study design, statistical analyses, and the drafted manuscript; Anita Visser contributed to the interpretation and critically reviewed the manuscript; Luc A.M. Marks contributed to the interpretation and critically reviewed the manuscript; and Geerten‐Has E. Tjakkes contributed to the study conception and design and data interpretation and critically reviewed the manuscript. All authors gave final approval and agreed to be held accountable for all aspects of this work to ensure integrity and accuracy.

## Supporting information


**Supplemental Figure S1** STROBE Statement—Checklist of items that should be included in reports of cohort studies

**Supplemental Figure S2** Flow chart of the participants included in the NHANES study (1999–2002)

**Supplemental Table S1** Healthy eating index–2015 components and scoring standards

**Supplemental Table S2** Categorization of cardiometabolic risk factors

**Supplemental Table S3.1‐3.3** Characteristics of study participants in NHANES 1999–2002 stratified by cognitive performance and caries status (*n* = 1,478)

**Supplemental** **Table S4** The association of cognitive impairment or oral health outcomes with cardiovascular disease risk factors

**Supplemental** **Table S5** The association of cognitive impairment and oral health outcomes with cardiometabolic mortality risk

**Supplemental Table S6** Sensitivity analyses for combined associations of cognitive impairment and EFP/AAP periodontal health with all‐cause and cardiometabolic mortality in the NHANES 2001–2002

**Supplemental Table S7** Sensitivity analyses for combined associations of cognitive impairment and severe tooth loss with all‐cause and cardiometabolic mortality in the NHANES 1999–2002

Supplemental Methods
