# Supplementary material for: Combined association of cognitive impairment and poor oral health on mortality risk in older adults: Results from the NHANES with 15 years of follow‐up
Source: J Periodontol. 2021 Nov 12;93(6):888–900. doi: 10.1002/JPER.21-0292 (PMC9298999; doi:10.1002/JPER.21-0292)
Supplement: Supplementary file 1 — Supplemental Figure S1 STROBE Statement—Checklist of items that should be included in reports of cohort studies [file JPER-93-888-s004.pdf]

Supplemental Figure S1

STROBE Statement—Checklist of items that should be included in reports of ***cohort studies***

	Item No	Recommendation	Yes/No/NA	Page/Line
**Title and abstract**	1	(*a*) Indicate the study's design with a commonly used term in the title or the abstract	Yes	Page 3
		(*b*) Provide in the abstract an informative and balanced summary of what was done and what was found	Yes	Page 3
**Introduction**				
Background/rationale	2	Explain the scientific background and rationale for the investigation being reported	Yes	Page 4
Objectives	3	State specific objectives, including any prespecified hypotheses	Yes	Page 5
**Methods**				
Study design	4	Present key elements of study design early in the paper	Yes	Page 5
Setting	5	Describe the setting, locations, and relevant dates, including periods of recruitment, exposure, follow-up, and data collection	Yes	Page 5
Participants	6	(*a*) Give the eligibility criteria, and the sources and methods of selection of participants. Describe methods of follow-up	Yes	Page 10
		(*b*) For matched studies, give matching criteria and number of exposed and unexposed	Yes	Page 6-8
Variables	7	Clearly define all outcomes, exposures, predictors, potential confounders, and effect modifiers. Give diagnostic criteria, if applicable	Yes	Page 8
Data sources/ measurement	8*	For each variable of interest, give sources of data and details of methods of assessment (measurement). Describe comparability of assessment methods if there is more than one group	Yes	Page 8
Bias	9	Describe any efforts to address potential sources of bias	NA	
Study size	10	Explain how the study size was arrived at	Yes	Page 10
Quantitative variables	11	Explain how quantitative variables were handled in the analyses. If applicable, describe which groupings were chosen and why	Yes	Page 7
Statistical methods	12	(*a*) Describe all statistical methods, including those used to control for confounding	Yes	Page 9
		(*b*) Describe any methods used to examine subgroups and interactions	NA	
		(*c*) Explain how missing data were addressed	Yes	Page 10
		(*d*) If applicable, explain how loss to follow-up was addressed	NA	
		(*e*) Describe any sensitivity analyses	Yes	Page 10
**Results**				
Participants	13*	(a) Report numbers of individuals at each stage of study—eg numbers potentially eligible, examined for	Yes	Page 10

		eligibility, confirmed eligible, included in the study, completing follow-up, and analysed		
		(b) Give reasons for non-participation at each stage	Yes	Page 10
		(c) Consider use of a flow diagram	Yes	Appendix Figure 1
Descriptive data	14*	(a) Give characteristics of study participants (eg demographic, clinical, social) and information on exposures and potential confounders	Yes	Page 10
		(b) Indicate number of participants with missing data for each variable of interest	NA	
		(c) Summarise follow-up time (eg, average and total amount)	Yes	Page 10
Outcome data	15*	Report numbers of outcome events or summary measures over time	Yes	Page 11
Main results	16	(*a*) Give unadjusted estimates and, if applicable, confounder-adjusted estimates and their precision (eg, 95% confidence interval). Make clear which confounders were adjusted for and why they were included	Yes	Page 12
		(*b*) Report category boundaries when continuous variables were categorized	Yes	Appendix Table 1
		(*c*) If relevant, consider translating estimates of relative risk into absolute risk for a meaningful time period	Yes	Page 11
Other analyses	17	Report other analyses done—eg analyses of subgroups and interactions, and sensitivity analyses	Yes	Appendix Table 4, 5
**Discussion**				
Key results	18	Summarise key results with reference to study objectives	Yes	Page 12
Limitations	19	Discuss limitations of the study, taking into account sources of potential bias or imprecision. Discuss both direction and magnitude of any potential bias	Yes	Page 14
Interpretation	20	Give a cautious overall interpretation of results considering objectives, limitations, multiplicity of analyses, results from similar studies, and other relevant evidence	Yes	Page 12
Generalisability	21	Discuss the generalisability (external validity) of the study results	Yes	Page 13
**Other information**				
Funding	22	Give the source of funding and the role of the funders for the present study and, if applicable, for the original study on which the present article is based	Yes	Page 15

*Give information separately for exposed and unexposed groups.

Note: An Explanation and Elaboration article discusses each checklist item and gives methodological background and published examples of transparent reporting. The STROBE checklist is best used in conjunction with this article (freely available on the Web sites of PLoS Medicine at http://www.plosmedicine.org/, Annals of Internal Medicine at http://www.annals.org/, and Epidemiology at http://www.epidem.com/). Information on the STROBE Initiative is available at http://www.strobe-statement.org.
