# Supplementary figures and images for: Combined association of cognitive impairment and poor oral health on mortality risk in older adults: Results from the NHANES with 15 years of follow‐up
Source: J Periodontol. 2021 Nov 12;93(6):888–900. doi: 10.1002/JPER.21-0292 (PMC9298999; doi:10.1002/JPER.21-0292)

## Supplemental Figure S2

Flow chart of the participants included in the NHANES study (1999–2002).

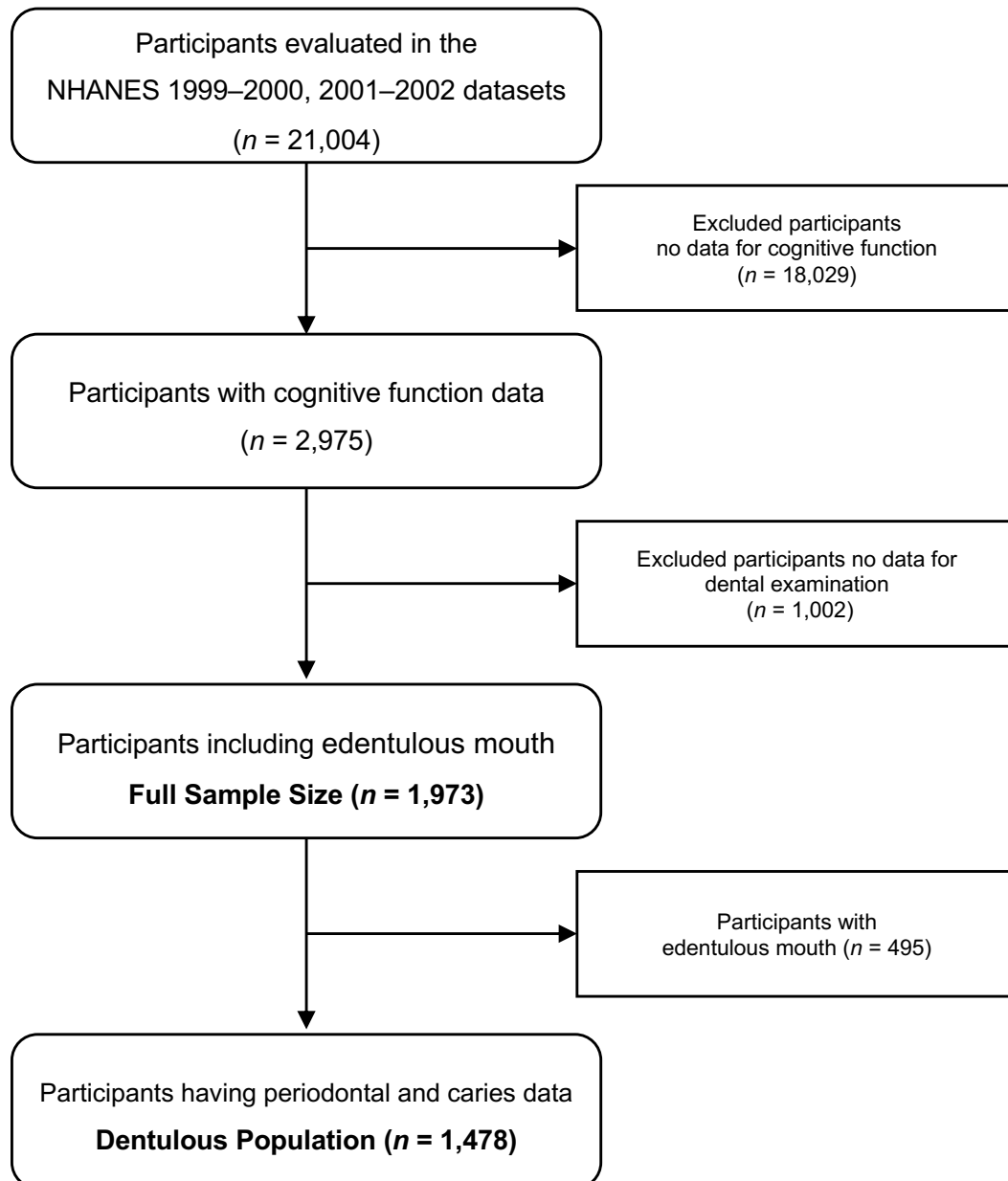

Supplement: Supplementary file 2 — Supplemental Figure S2 Flow chart of the participants included in the NHANES study (1999–2002) [file JPER-93-888-s009.pdf]
