# Supplementary material for: Combined association of cognitive impairment and poor oral health on mortality risk in older adults: Results from the NHANES with 15 years of follow‐up
Source: J Periodontol. 2021 Nov 12;93(6):888–900. doi: 10.1002/JPER.21-0292 (PMC9298999; doi:10.1002/JPER.21-0292)
Supplement: Supplementary file 3 — Supplemental Table S1 Healthy eating index–2015 components and scoring standards [file JPER-93-888-s002.docx]

**Supplemental Table *S*1** Healthy eating index–2015 components and scoring standards

| HEI-2015 | Maximum | Standard for maximum score | Standard for minimum score |
| --- | --- | --- | --- |
| **Adequacy components** |  |  |  |
| Total fruits | 0 to 5 | > 0.8 cup equiv. /1,000kcal | No fruit |
| Whole fruits | 0 to 5 | > 0.4 cup equiv. /1,000kcal | No whole fruit |
| Total vegetables | 0 to 5 | > 1.1 cup equiv. /1,000kcal | No vegetable |
| Greens and beans | 0 to 5 | > 0.2 cup equiv. /1,000kcal | No dark-green vegetables |
| Whole grains | 0 to 10 | > 1.5 ounce equiv. /1,000kcal | No whole grains |
| Dairy | 0 to 10 | > 1.3 cup equiv. /1,000kcal | No dairy |
| Total protein foods | 0 to 5 | > 2.5 ounce equiv. /1,000kcal | No protein foods |
| Seafood and plant proteins | 0 to 5 | > 0.8 ounce equiv. /1,000kcal | No seafood or plant proteins |
| Fatty acid | 0 to 10 | (PUFAs + MUFAs)/SFAs >2.5 | (PUFAs + MUFAs)/SFAs <1.2 |
| **Moderation components** |  |  |  |
| Refined grains | 10 to 0 | < 1.8 ounce equiv. /1,000 kcal | > 4.3 ounce equiv. /1,000 kcal |
| Sodium | 10 to 0 | < 1.1g/1,000 kcal | > 2.0 g / 1,000 kcal |
| Added sugars | 10 to 0 | < 6.5% of energy | > 26% of energy |
| Saturated fats | 10 to 0 | < 8% of energy | > 16% of energy |
| **Total score** | 0 to 100 | - | **-** |
