# Supplementary material for: Combined association of cognitive impairment and poor oral health on mortality risk in older adults: Results from the NHANES with 15 years of follow‐up
Source: J Periodontol. 2021 Nov 12;93(6):888–900. doi: 10.1002/JPER.21-0292 (PMC9298999; doi:10.1002/JPER.21-0292)
Supplement: Supplementary file 4 — Supplemental Table S2 Categorization of cardiometabolic risk factors [file JPER-93-888-s008.docx]

**Supplemental Table *S*2** Categorization of Cardiometabolic Risk Factors

| **Cardiovascular Disease Risk Factors** | | |
| --- | --- | --- |
|  | Measurement, Units | 1. Categorical Definitions |
| Obesity | Body mass index (BMI), calculated as weight in kilograms divided by height in meters squared, kg/m^2^ | 1. Normal is defined as BMI of <25 kg/m^2^; Overweight is defined as BMI of ≥25 to <30 kg/m^2^; 2. Obesity is defined as BMI of at least 30 kg/m^2^. |
| Hypertension | Systolic blood pressure (SBP), mm Hg and diastolic blood pressure (DBP), mm Hg; Self-reported physician's diagnosis | 1. Normal is no self-reported physician's diagnosis and SBP < 120 mm Hg and DBP < 80 mm Hg; Prehypertension is no self-reported physician's diagnosis and SBP is 120 to 140 mm Hg or DBP is 80 to 90 mm Hg; 2. Hypertension is defined as diagnosed (self-reported) or undiagnosed (no self-reported diagnosis and SBP >140 mm Hg or DBP >90 mm Hg) hypertension or currently taking antihypertensive medication. |
| Dyslipidemia | Non-HDL (high-density lipoprotein) cholesterol level, mg/dL; Self-reported physician's diagnosis | 1. Normal is no self-reported physician's diagnosis and non-HDL cholesterol level < 130 mg/dL; Intermediate dyslipidemia is no self-reported physician's diagnosis and non-HDL cholesterol level is 130 to 160 mg/dL; 2. Dyslipidemia is defined as diagnosed (self-reported) or undiagnosed (no self-reported diagnosis and non-HDL cholesterol level >160 mg/dL) dyslipidemia or currently taking anticholesterolemia medication. |
| Diabetes mellitus | Glycated hemoglobin level, %; Self-reported physician's diagnosis | 1. Normal is no self-reported physician's diagnosis and glycated hemoglobin level < 5.7%; Intermediate dysglycemia is no self-reported physician's diagnosis and glycated hemoglobin level >5.7%; 2. Diabetes mellitus is defined as self-reported diabetes diagnosis or glycated hemoglobin level of at least 5.7%. |
| **Medical Conditions** | | |
|  | Measurement, Units | Categorical Definitions |
| Elevated C-reactive protein level | High-sensitivity C-reactive protein (CRP) level, mg/dL | 1. Tertile 1 is 0.01 to 0.16 mg/dL, tertile 2 is >0.16 to <0.40 mg/dL, tertile 3 is >0.40 mg/dL; 2. Elevated C-reactive protein level is defined as CRP of at least 0.64 mg/dL. |
| Heart disease | Self-reported physician's diagnosis | 1. Normal is no self-reported physician's diagnosis; 2. Heart disease is self-reported physician's diagnosis of congestive heart failure or coronary heart disease or angina pectoris or heart attack. |
| Stroke | Self-reported physician's diagnosis | 1. Normal is no self-reported physician's diagnosis;  2. Stroke is self-reported physician's diagnosis of stroke. |
