# Supplementary material for: Combined association of cognitive impairment and poor oral health on mortality risk in older adults: Results from the NHANES with 15 years of follow‐up
Source: J Periodontol. 2021 Nov 12;93(6):888–900. doi: 10.1002/JPER.21-0292 (PMC9298999; doi:10.1002/JPER.21-0292)
Supplement: Supplementary file 5 — Supplemental Table S3.1‐3.3 Characteristics of study participants in NHANES 1999–2002 stratified by cognitive performance and caries status (n = 1,478) [file JPER-93-888-s006.docx]

**Supplemental Table *S*3.1** Characteristics of study participants in NHANES 1999–2002 stratified by cognitive performance and caries status (*n* = 1,478) ^*^

| COVARIATES |  | Normal cognition | | Cognitive impairment | | *P*  Value ^‡^ |
| --- | --- | --- | --- | --- | --- | --- |
|  | All | No untreated caries | Untreated caries | No untreated caries | Untreated caries |  |
|  | (*n* = 1,478) | (*n* = 941) | (*n* = 207) | (*n* = 209) | (*n* = 121) |  |
| Continuous Variables, Mean (SD) |  |  |  |  |  |  |
| Age (year) | 69.82 (7.51) | 69.55 (7.32) | 68.81 (7.30) | 71.63 (7.88) | 70.48 (8.11) | < .001 |
| Body mass index (kg/m^2^) | 28.27 (5.21) | 28.05 (5.08) | 29.24 (5.59) | 28.16 (5.24) | 28.61 (5.37) | .027 |
| Healthy eating index-2015 | 54.13 (11.57) | 54.27 (11.82) | 53.04 (11.24) | 54.38 (11.70) | 53.47 (11.62) | .508 |
| Systolic blood pressure (mmHg) | 138.68 (21.19) | 137.85 (20.30) | 137.37 (21.31) | 139.28 (23.43) | 146.45 (22.33) | < .001 |
| Diastolic blood pressure (mmHg) | 70.25 (14.96) | 70.68 (14.14) | 70.46 (16.22) | 68.09 (14.03) | 70.18 (19.79) | .174 |
| Non-HDL cholesterol (mg/dL) | 158.78 (39.41) | 159.21 (37.99) | 160.15 (43.59) | 156.07 (39.58) | 157.54 (43.01) | .718 |
| Glycohemoglobin (%) | 5.87 (1.14) | 5.74 (0.91) | 6.21 (1.70) | 6.04 (1.19) | 6.07 (1.35) | < .001 |
| Categorical Variables, n (%) |  |  |  |  |  |  |
| Male | 763 (51.6) | 454 (48.2) | 120 (58.0) | 115 (55.0) | 74 (61.2) | .005 |
| Race/ethnicity |  |  |  |  |  |  |
| Non-Hispanic white | 836 (56.6) | 649 (69.0) | 104 (50.2) | 59 (28.2) | 24 (19.8) | < .001 |
| Non-Hispanic black | 220 (14.9) | 100 (10.6) | 41 (19.8) | 46 (22.0) | 33 (27.3) |  |
| Other race, including multiracial | 422 (28.6) | 192 (20.4) | 62 (30.0) | 104 (49.8) | 64 (52.9) |  |
| Education level ^†^ |  |  |  |  |  |  |
| <high school | 882 (59.7) | 454 (48.3) | 134 (64.7) | 183 (87.6) | 111 (92.5) | < .001 |
| college | 316 (21.4) | 246 (26.2) | 48 (23.2) | 16 (7.7) | 6 (5.0) |  |
| >college | 278 (18.8) | 240 (25.5) | 25 (12.1) | 10 (4.8) | 3 (2.5) |  |
| Annual household income ^†^ |  |  |  |  |  |  |
| <20,000$ | 480 (32.5) | 214 (23.5) | 85 (42.1) | 105 (53.6) | 76 (63.9) | < .001 |
| 20,000-75,000$ | 787 (53.2) | 554 (60.8) | 109 (54.0) | 83 (42.3) | 41 (34.5) |  |
| >75,000$ | 161 (10.9) | 143 (15.7) | 8 (4.0) | 8 (4.1) | 2 (1.7) |  |
| Smoking habit ^†^ |  |  |  |  |  |  |
| Non smoker | 727 (49.2) | 469 (50.0) | 85 (41.1) | 103 (49.3) | 70 (57.9) | .001 |
| Former smoker | 599 (40.5) | 392 (41.8) | 86 (41.5) | 85 (40.7) | 36 (29.8) |  |
| Current smoker | 149 (10.1) | 77 (8.2) | 36 (17.4) | 21 (10.0) | 15 (12.4) |  |
| Alcohol intake > 12 drinks/year ^†^ | 922 (62.4) | 615 (66.3) | 128 (63.1) | 115 (56.4) | 64 (55.7) | .014 |
| Time since the last dental visit ^†^ |  |  |  |  |  |  |
| Less than 1 year | 955 (64.6) | 723 (76.8) | 77 (37.4) | 123 (59.1) | 32 (26.9) | < .001 |
| 1-3 years | 251 (17.0) | 125 (13.3) | 56 (27.2) | 37 (17.8) | 33 (27.7) |  |
| More than 3 years | 268 (18.1) | 93 (9.9) | 73 (35.4) | 48 (23.1) | 54 (45.4) |  |
| Obesity ^†^ | 451 (30.5) | 274 (29.4) | 79 (38.7) | 63 (31.2) | 35 (31.0) | .080 |
| Elevated C-reactive protein level ^†^ | 453 (30.6) | 285 (31.0) | 65 (33.3) | 60 (31.4) | 43 (37.7) | .515 |
| Hypertension ^†^ | 681 (46.1) | 424 (45.3) | 96 (47.1) | 98 (47.1) | 63 (52.5) | .499 |
| Dyslipidemia ^†^ | 681 (46.1) | 436 (47.2) | 94 (46.5) | 96 (49.2) | 55 (48.2) | .946 |
| Diabetes mellitus ^†^ | 231 (15.6) | 109 (11.9) | 50 (24.6) | 44 (21.4) | 28 (23.3) | < .001 |
| Heart disease ^†^ | 184 (12.5) | 103 (11.0) | 24 (11.7) | 40 (19.3) | 17 (14.2) | .011 |
| Stroke | 71 (4.8) | 35 (3.7) | 9 (4.4) | 19 (9.1) | 8 (6.6) | .009 |

^*^ Cognitive impairment defined as DSST score <29 (the lowest quartile of score in population); Untreated caries was defined as having any decayed permanent tooth surfaces.

^†^ Missing values for total study: education (*n* = 2; <1%), income (*n* = 50; 3.4%), smoking (*n* = 3; <1%), alcohol (*n* = 28; 1.9%), dental visit (*n* = 4; <1%), hypertension (*n* = 3; <1%), diabetes (*n* = 36; 2.4%), obesity (*n* = 28; 1.9%), abdominal adiposity (*n* = 56; 3.8%), elevated C-reactive protein level (*n* = 60; 4.1%), dyslipidemia (*n* = 43; 2.9%), heart disease (*n* = 7; <1%) and stroke (*n* = 4; <1%).

^‡^ *P* Value by one-way ANOVA for continuous variables and by χ^2^ test for categorical values.

Abbreviations: SD, standard deviation; HDL, high-density lipoprotein; DSST, digit symbol substitution test.

**Supplemental Table *S*3.2** Characteristics of study participants in NHANES 1999–2002 stratified by cognitive performance and periodontal status (*n* = 1,478) ^*^

| COVARIATES |  | Normal cognition | | Cognitive impairment | | *P*  Value ^‡^ |
| --- | --- | --- | --- | --- | --- | --- |
|  | All | No/mild  periodontitis | Moderate/severe periodontitis | No/mild  periodontitis | Moderate/severe periodontitis |  |
|  | (*n* = 1,478) | (*n* = 597) | (*n* = 506) | (*n* = 149) | (*n* = 226) |  |
| Continuous Variables, Mean (SD) |  |  |  |  |  |  |
| Age (year) | 69.82 (7.51) | 68.62 (7.15) | 70.20 (7.39) | 71.11 (7.86) | 71.27 (7.98) | < .001 |
| Body mass index (kg/m^2^) | 28.27 (5.21) | 28.59 (5.16) | 27.93 (5.29) | 28.38 (4.95) | 28.14 (5.32) | .221 |
| Healthy eating index-2015 | 54.13 (11.57) | 54.29 (11.67) | 53.72 (11.70) | 52.93 (12.23) | 54.89 (11.47) | .365 |
| Systolic blood pressure (mmHg) | 138.68 (21.19) | 137.07 (20.04) | 138.25 (20.69) | 143.01 (23.54) | 141.13 (23.08) | .006 |
| Diastolic blood pressure (mmHg) | 70.25 (14.96) | 71.03 (13.99) | 70.05 (15.25) | 67.86 (15.94) | 70.16 (16.06) | .153 |
| Non-HDL cholesterol (mg/dL) | 158.78 (39.41) | 162.29 (40.41) | 156.11 (37.31) | 155.04 (43.15) | 157.75 (38.32) | .042 |
| Glycohemoglobin (%) | 5.87 (1.14) | 5.76 (0.93) | 5.86 (1.20) | 5.93 (1.07) | 6.16 (1.45) | < .001 |
| Categorical Variables, *n* (%) |  |  |  |  |  |  |
| Male | 763 (51.6) | 264 (44.2) | 281 (55.5) | 72 (48.3) | 146 (64.6) | < .001 |
| Race/ethnicity |  |  |  |  |  |  |
| Non-Hispanic white | 836 (56.6) | 413 (69.2) | 321 (63.4) | 39 (26.2) | 63 (27.9) | < .001 |
| Non-Hispanic black | 220 (14.9) | 68 (11.4) | 63 (12.5) | 34 (22.8) | 55 (24.3) |  |
| Other race, including multiracial | 422 (28.6) | 116 (19.4) | 122 (24.1) | 76 (51.0) | 108 (47.8) |  |
| Education level ^†^ |  |  |  |  |  |  |
| <high school | 882 (59.7) | 272 (45.6) | 278 (55.0) | 124 (83.2) | 208 (92.4) | < .001 |
| college | 316 (21.4) | 157 (26.3) | 132 (26.1) | 19 (12.8) | 8 (3.6) |  |
| >college | 278 (18.8) | 168 (28.1) | 95 (18.8) | 6 (4.0) | 9 (4.0) |  |
| Annual household income ^†^ |  |  |  |  |  |  |
| <20,000$ | 480 (32.5) | 119 (20.5) | 157 (32.0) | 64 (45.7) | 140 (64.5) | < .001 |
| 20,000-75,000$ | 787 (53.2) | 356 (61.4) | 288 (58.7) | 72 (51.4) | 71 (32.7) |  |
| >75,000$ | 161 (10.9) | 105 (18.1) | 46 (9.4) | 4 (2.9) | 6 (2.8) |  |
| Smoking habit ^†^ |  |  |  |  |  |  |
| Non smoker | 727 (49.2) | 323 (54.1) | 212 (42.1) | 91 (61.1) | 101 (44.9) | < .001 |
| Former smoker | 599 (40.5) | 236 (39.5) | 223 (44.2) | 47 (31.5) | 93 (41.3) |  |
| Current smoker | 149 (10.1) | 38 (6.4) | 69 (13.7) | 11 (7.4) | 31 (13.8) |  |
| Alcohol intake > 12 drinks/year ^†^ | 922 (62.4) | 380 (64.6) | 336 (67.5) | 73 (51.0) | 133 (60.2) | .002 |
| Time since the last dental visit ^†^ |  |  |  |  |  |  |
| Less than 1 year | 955 (64.6) | 448 (75.2) | 328 (64.8) | 86 (57.7) | 93 (41.7) | < .001 |
| 1-3 years | 251 (17.0) | 81 (13.6) | 90 (17.8) | 30 (20.1) | 50 (22.4) |  |
| More than 3 years | 268 (18.1) | 67 (11.2) | 88 (17.4) | 33 (22.1) | 80 (35.9) |  |
| Obesity ^†^ | 451 (30.5) | 191 (32.3) | 151 (30.2) | 46 (32.2) | 63 (29.2) | .789 |
| Elevated C-reactive protein level ^†^ | 453 (30.6) | 196 (33.7) | 138 (28.3) | 42 (30.0) | 77 (36.8) | .093 |
| Hypertension ^†^ | 681 (46.1) | 265 (44.7) | 231 (45.9) | 74 (50.0) | 111 (49.3) | .514 |
| Dyslipidemia ^†^ | 681 (46.1) | 290 (49.2) | 218 (44.3) | 63 (45.0) | 110 (51.4) | .223 |
| Diabetes mellitus ^†^ | 231 (15.6) | 77 (13.3) | 73 (14.8) | 31 (21.1) | 50 (22.4) | .004 |
| Heart disease ^†^ | 184 (12.5) | 62 (10.4) | 54 (10.7) | 27 (18.1) | 41 (18.4) | .002 |
| Stroke | 71 (4.8) | 20 (3.4) | 20 (4.0) | 16 (10.8) | 15 (6.6) | .001 |

^*^ Cognitive impairment defined as DSST score <29 (the lowest quartile of score in population); Moderate/severe periodontitis was defined by the CDC/AAP case definition.

^†^ Missing values for total study: education (*n* = 2; <1%), income (*n* = 50; 3.4%), smoking (*n* = 3; <1%), alcohol (*n* = 28; 1.9%), dental visit (*n* = 4; <1%), hypertension (*n* = 3; <1%), diabetes (*n* = 36; 2.4%), obesity (*n* = 28; 1.9%), abdominal adiposity (*n* = 56; 3.8%), elevated C-reactive protein level (*n* = 60; 4.1%), dyslipidemia (*n* = 43; 2.9%), heart disease (*n* = 7; <1%) and stroke (*n* = 4; <1%).

^‡^ *P* Value by one-way ANOVA for continuous variables and by χ^2^ test for categorical values.

Abbreviations: SD, standard deviation; HDL, high-density lipoprotein; DSST, digit symbol substitution test.

**Supplemental Table *S*3.3** Characteristics of study participants in NHANES 1999–2002 stratified by cognitive performance and dentate status (*n* = 1,973) ^*^

|  |  | Normal cognition | | Cognitive impairment | | *P*  Value ^‡^ |
| --- | --- | --- | --- | --- | --- | --- |
|  | All | Dentulous population | Edentulous population | Dentulous population | Edentulous population |  |
| **Variables** | (*n* = 1,973) | (*n* = 1,148) | (*n* = 316) | (*n* = 330) | (*n* = 179) |  |
| Continuous Variables, Mean (SD) |  |  |  |  |  |  |
| Age (year) | 70.68 (7.70) | 69.41 (7.32) | 72.27 (7.45) | 71.21 (7.97) | 74.98 (7.84) | < .001 |
| Body mass index (kg/m^2^) | 28.19 (5.27) | 28.26 (5.19) | 28.04 (5.45) | 28.32 (5.29) | 27.75 (5.52) | .623 |
| Healthy eating index-2015 | 54.13 (11.57) | 54.05 (11.72) | 54.66 (11.31) | 54.05 (11.67) | 53.92 (10.86) | .850 |
| Systolic blood pressure (mmHg) | 139.68 (21.88) | 137.76 (20.48) | 139.87 (22.62) | 141.93 (23.26) | 147.59 (24.52) | < .001 |
| Diastolic blood pressure (mmHg) | 69.73 (15.13) | 70.64 (14.53) | 67.76 (15.79) | 68.85 (16.36) | 68.84 (15.15) | .012 |
| Non-HDL cholesterol (mg/dL) | 160.14(39.77) | 159.37 (39.02) | 165.08 (41.20) | 156.61 (40.83) | 162.92 (39.63) | .044 |
| Glycohemoglobin (%) | 5.90 (1.16) | 5.82 (1.11) | 5.91 (1.13) | 6.05 (1.25) | 6.12 (1.35) | .001 |
| Categorical Variables, *n* (%) |  |  |  |  |  |  |
| Male | 987 (50.0) | 574 (50.0) | 137 (43.4) | 189 (57.3) | 87 (48.6) | .005 |
| Race/ethnicity |  |  |  |  |  |  |
| Non-Hispanic white | 1148 (58.2) | 753 (65.6) | 229 (72.5) | 83 (25.2) | 83 (46.4) | < .001 |
| Non-Hispanic black | 307 (15.6) | 141 (12.3) | 42 (13.3) | 79 (23.9) | 45 (25.1) |  |
| Other race, including multiracial | 518 (26.3) | 254 (22.1) | 45 (14.2% | 168 (50.9% | 51 (28.5% |  |
| Education level ^†^ |  |  |  |  |  |  |
| <high school | 1287 (65.3) | 588 (51.3) | 244 (77.2) | 294 (89.4) | 161 (90.4) | < .001 |
| college | 390 (19.8) | 294 (25.6) | 61 (19.3) | 22 (6.7) | 13 (7.3) |  |
| >college | 293 (14.9) | 265 (23.1) | 11 (3.5) | 13 (4.0) | 4 (2.2) |  |
| Annual household income ^†^ |  |  |  |  |  |  |
| <20,000$ | 745 (39.3) | 299 (26.9) | 144 (48.6) | 181 (57.5) | 121 (70.8) | < .001 |
| 20,000-75,000$ | 971 (51.2) | 663 (59.6) | 135 (45.6) | 124 (39.4) | 49 (28.7) |  |
| >75,000$ | 179 (9.4) | 151 (13.6) | 17 (5.7) | 10 (3.2) | 1 (0.6) |  |
| Smoking habit ^†^ |  |  |  |  |  |  |
| Non smoker | 909 (46.2) | 554 (48.4) | 110 (34.8) | 173 (52.4) | 72 (40.4) | < .001 |
| Former smoker | 805 (40.9) | 478 (41.7) | 134 (42.4) | 121 (36.7) | 72 (40.4) |  |
| Current smoker | 255 (13.0) | 113 (9.9) | 72 (22.8) | 36 (10.9% | 34 (19.1% |  |
| Alcohol intake > 12 drinks/year ^†^ | 1183 (61.2) | 743 (65.7) | 171 (55.3) | 179 (56.1) | 90 (51.7) | < .001 |
| Time since the last dental visit ^†^ |  |  |  |  |  |  |
| Less than 1 year | 1,040 (52.8) | 800 (69.7) | 53 (16.8) | 155 (47.4) | 32 (18.0) | < .001 |
| 1-3 years | 320 (16.3) | 181 (15.8) | 44 (13.9) | 70 (21.4) | 25 (14.0) |  |
| More than 3 years | 608 (30.9) | 166 (14.5) | 219 (69.3) | 102 (31.2) | 121 (68.0) |  |
| Obesity ^†^ | 600 (31.2) | 353 (31.1) | 99 (32.6) | 98 (31.1) | 50 (29.9) | .942 |
| Elevated C-reactive protein level ^†^ | 636 (33.8) | 350 (31.4) | 111 (37.4) | 103 (33.8) | 72 (42.9) | .014 |
| Hypertension ^†^ | 942 (48.0) | 520 (45.6) | 164 (51.9) | 161 (49.1) | 97 (54.5) | .051 |
| Dyslipidemia ^†^ | 945 (49.5) | 530 (47.1) | 171 (56.1) | 151 (48.9) | 93 (54.7) | .020 |
| Diabetes mellitus ^†^ | 328 (17.0) | 159 (14.2) | 50 (16.2) | 72 (22.1) | 47 (27.0) | < .001 |
| Heart disease ^†^ | 306 (15.6) | 127 (11.1) | 77 (24.7) | 57 (17.4) | 45 (25.7) | .000 |
| Stroke | 115 (5.8) | 44 (3.8) | 21 (6.7) | 27 (8.2) | 23 (12.9) | .000 |

^*^ Cognitive impairment defined as DSST score <29 (the lowest quartile of score in population); Edentulism was defined as the complete loss of all-natural teeth.

^†^ Missing values for total study: education (n = 3; <1%), income (n = 78; 4.0%), smoking (n = 4; <1%), alcohol (n = 40; 2.0%), dental visit (n = 5; <1%), hypertension (n = 3; <1%), diabetes (n = 30; 3%), obesity (n = 52; 2.6%), abdominal adiposity (n = 96; 4.9%), elevated C-reactive protein level (n = 90; 4.6%), dyslipidemia (n = 63; 3.2%), heart disease (n = 15; <1%), and stroke (n = 6; <1%).

^‡^ *P* Value by one-way ANOVA for continuous variables and by χ^2^ test for categorical values.

Abbreviations: SD, standard deviation; HDL, high-density lipoprotein; DSST, digit symbol substitution test.
