# Supplementary material for: Combined association of cognitive impairment and poor oral health on mortality risk in older adults: Results from the NHANES with 15 years of follow‐up
Source: J Periodontol. 2021 Nov 12;93(6):888–900. doi: 10.1002/JPER.21-0292 (PMC9298999; doi:10.1002/JPER.21-0292)
Supplement: Supplementary file 6 — Supplemental Table S4 The association of cognitive impairment or oral health outcomes with cardiovascular disease risk factors [file JPER-93-888-s010.docx]

**Table *S*4** The association of cognitive impairment or oral health outcomes with cardiovascular disease risk factors

| Odds Ratio (95% CI) | Cardiovascular Disease Risk Factors | | | |
| --- | --- | --- | --- | --- |
|  | 1 vs 0 | 2 vs 0 | 3 vs 0 | 4 vs 0 |
| Cognitive Impairment |  |  |  |  |
| Unadjusted | **1.87**  **(1.32 to 2.65)** | **2.02**  **(1.41 to 2.89)** | **3.15**  **(2.08 to 4.77)** | **6.29**  **(2.94 to 13.47)** |
| Adjusted | **1.65**  **(1.08 to 2.53)** | **1.68**  **(1.08 to 2.61)** | **2.19**  **(1.29 to 3.72)** | **7.66**  **(2.82 to 20.83)** |
| Untreated Caries |  |  |  |  |
| Unadjusted | 1.04  (0.71 to 1.51) | 1.37  (0.93 to 2.01) | **1.82**  **(1.14 to 2.90)** | **2.98**  **(1.20 to 7.38)** |
| Adjusted | 0.89  (0.58 to 1.39) | 1.06  (0.68 to 1.67) | 1.26  (0.71 to 2.22) | 1.95  (0.61 to 6.23) |
| Moderate/Severe Periodontitis | |  |  |  |
| Unadjusted | 1.03  (0.77 to 1.39) | 1.17  (0.86 to 1.60) | **1.71**  **(1.14 to 2.56)** | **3.11**  **(1.18 to 8.20)** |
| Adjusted | 1.08  (0.77 to 1.51) | 1.32  (0.93 to 1.89) | 1.54  (0.96 to 2.46) | **4.00**  **(1.25 to 12.77)** |
| Edentulous Population | |  |  |  |
| Unadjusted | 1.30  (0.94 to 1.80) | **1.68**  **(1.20 to 2.34)** | **1.68**  **(1.12 to 2.53)** | 1.92  (0.86 to 4.27) |
| Adjusted | 1.52  (0.99 to 2.31) | **1.60**  **(1.04 to 2.46)** | 1.49  (0.88 to 2.53) | 1.02  (0.35 to 2.95) |

^a^ Cardiovascular disease risk factors included obesity, hypertension, dyslipidemia, and diabetes mellitus.

^b^ The multinominal log-linear model was adjusted for sociodemographic variables (age, gender, race/ethnicity, educational level, and income level), behavioral variables (diet quality, smoking, drinking, and dental visit), and medical conditions (elevated systemic inflammation, heart disease, and stroke).

Boldface indicates statistical significance (*p* value <0.05).
