# Supplementary material for: Combined association of cognitive impairment and poor oral health on mortality risk in older adults: Results from the NHANES with 15 years of follow‐up
Source: J Periodontol. 2021 Nov 12;93(6):888–900. doi: 10.1002/JPER.21-0292 (PMC9298999; doi:10.1002/JPER.21-0292)
Supplement: Supplementary file 7 — Supplemental Table S5 The association of cognitive impairment and oral health outcomes with cardiometabolic mortality risk [file JPER-93-888-s007.docx]

**Table *S*5** The association of cognitive impairment and oral health outcomes with cardiometabolic mortality risk ^*^

| Exposure Variables | Crude Model | | Adjusted Model ^†^ | |
| --- | --- | --- | --- | --- |
|  | Hazard Ratio | 95% CI | Hazard Ratio | 95% CI |
| Cognitive Performance |  |  |  |  |
| normal cognition | 1 [reference] | | 1 [reference] | |
| cognitive impairment | **2.305** | **(1.770 to 3.001)** | **1.477** | **(1.086 to 2.007)** |
| Caries Status |  |  |  |  |
| no untreated caries | 1 [reference] | | 1 [reference] | |
| untreated caries | **1.807** | **(1.299 to 2.515)** | 1.228 | (0.872 to 1.729) |
| Periodontal Status |  |  |  |  |
| no/mild periodontitis | 1 [reference] | | 1 [reference] | |
| moderate/severe periodontitis | **2.019** | **(1.438 to 2.834)** | **1.411** | **(1.097 to 2.059)** |
| Dentate Status |  |  |  |  |
| dentulous population | 1 [reference] | | 1 [reference] | |
| edentulous population | **2.407** | **(1.846 to 3.139)** | 1.195 | (0.878 to 1.627) |

* Cardiometabolic mortality combined diseases of heart, cerebrovascular diseases, and diabetes mellitus.

† Multivariable Cox proportional hazards models were adjusted for sociodemographic variables, behavioral, clinical conditions, and cardiovascular disease risk factors (see legend of **Table 2**).

‡ Boldface indicates statistical significance (*p* value < 0.05).

Abbreviations: CI, confidence interval.
