# Supplementary material for: Combined association of cognitive impairment and poor oral health on mortality risk in older adults: Results from the NHANES with 15 years of follow‐up
Source: J Periodontol. 2021 Nov 12;93(6):888–900. doi: 10.1002/JPER.21-0292 (PMC9298999; doi:10.1002/JPER.21-0292)
Supplement: Supplementary file 8 — Supplemental Table S6 Sensitivity analyses for combined associations of cognitive impairment and EFP/AAP periodontal health with all‐cause and cardiometabolic mortality in the NHANES 2001–2002 [file JPER-93-888-s005.docx]

**Supplemental Table *S*6** Sensitivity Analyses for Combined associations of cognitive impairment and EFP/AAP periodontal health with all-cause and cardiometabolic mortality in the NHANES 2001–2002 ^*^

| All-Cause Mortality | | | |
| --- | --- | --- | --- |
| Combined Groups ^†^ | Cases/Participants | Crude Model HR (95% CI) | Adjusted Model HR (95% CI) ^‡^ |
| Group 1 | 46/189 | 1 [Reference] | 1 [Reference] |
| Group 2 | 160/421 | **1.821 (1.256 to 2.639)** | **1.503 (1.025 to 2.206)** |
| Group 3 | 15/32 | **2.334 (1.211 to 4.497)** | 1.582 (0.785 to 3.189) |
| Group 4 | 91/139 | **4.450 (2.958 to 6.696)** | **2.886 (1.769 to 4.709)** |
| *P* for interaction ^§^ |  | .900 | .605 |
| Cardiometabolic Mortality ^‖^ | | | |
| Combined Groups ^†^ | Cases/Participants | Crude Model HR (95% CI) | Adjusted Model HR (95% CI) ^‡^ |
| Group 1 | 9/189 | 1 [Reference] | 1 [Reference] |
| Group 2 | 40/421 | **2.280 (1.011 to 5.144)** | 1.680 (0.723 to 3.900) |
| Group 3 | 2/32 | 0.975 (0.120 to 7.923) | 0.538 (0.061 to 4.728) |
| Group 4 | 27/139 | **6.657 (2.811 to 15.766)** | **3.586 (1.271 to 10.119)** |
| *P* for interaction ^§^ |  | .321 | .219 |

^*^ Periodontal health was defined as no sites with PPD >3 mm and <10% of sites with BOP in an intact periodontium (CAL <3 mm); the others were set as a poor periodontal health.

^†^ Four combined groups included the following: Group 1: Normal cognition and periodontal health; Group 2: Normal cognition and poor periodontal health; Group 3: Cognitive impairment and periodontal health; Group 4: Cognitive impairment and poor periodontal health.

^‡^ Multivariable Cox proportional hazards models were adjusted for sociodemographic variables, behavioral, clinical conditions, and CVD-RF (see legend of **Table 2**).

^§^ In the interaction analyses, we included an interaction term (cognitive status * periodontal health status).

^‖^ Cardiometabolic mortality combined diseases of heart, cerebrovascular diseases, and diabetes mellitus. Boldface indicates statistical significance (*p* value < 0.05).

Abbreviations: HR, hazard ratio; CI, confidence interval; NHANES, National Health and Nutrition Examination Survey; CVD-RF, cardiovascular disease risk factors.
