# Supplementary material for: Combined association of cognitive impairment and poor oral health on mortality risk in older adults: Results from the NHANES with 15 years of follow‐up
Source: J Periodontol. 2021 Nov 12;93(6):888–900. doi: 10.1002/JPER.21-0292 (PMC9298999; doi:10.1002/JPER.21-0292)
Supplement: Supplementary file 9 — Supplemental Table S7 Sensitivity analyses for combined associations of cognitive impairment and severe tooth loss with all‐cause and cardiometabolic mortality in the NHANES 1999–2002 [file JPER-93-888-s001.docx]

**Supplemental Table *S*7** Sensitivity Analyses for Combined associations of cognitive impairment and severe tooth loss with all-cause and cardiometabolic mortality in the NHANES 1999–2002 ^*^

| All-Cause Mortality | | | |
| --- | --- | --- | --- |
| Combined Groups ^†^ | Cases/Participants | Crude Model HR (95% CI) | Adjusted Model HR (95% CI) ^‡^ |
| Group 1 | 359/995 | 1 [Reference] | 1 [Reference] |
| Group 2 | 288/469 | **2.147 (1.806 to 2.551)** | **1.423 (1.168 to 1.734)** |
| Group 3 | 141/268 | **1.692 (1.356 to 2.111)** | **1.401 (1.091 to 1.800)** |
| Group 4 | 190/241 | **3.218 (2.624 to 3.948)** | **1.841 (1.437 to 2.359)** |
| *P* for interaction ^§^ |  | .440 | .618 |
| Cardiometabolic Mortality ^‖^ | | | |
| Combined Groups ^†^ | Cases/Participants | Crude Model HR (95% CI) | Adjusted Model HR (95% CI) ^‡^ |
| Group 1 | 82/995 | 1 [Reference] | 1 [Reference] |
| Group 2 | 80/469 | **2.580 (1.838 to 3.621)** | **1.548 (1.050 to 2.283)** |
| Group 3 | 45/268 | **2.296 (1.522 to 3.463)** | 1.535 (0.959 to 2.457) |
| Group 4 | 57/241 | **3.529 (2.347 to 5.305)** | **1.705 (1.049 to 2.771)** |
| *P* for interaction ^§^ |  | .081 | .276 |

^*^ Severe tooth loss was defined as individuals with eight or less teeth.

^†^ Four combined groups included the following: Group 1: Normal cognition and number of teeth present >9; Group 2: Normal cognition and number of teeth present <8; Group 3: Cognitive impairment and number of teeth present >9; Group 4: Cognitive impairment and number of teeth present <8.

^‡^ Multivariable Cox proportional hazards models were adjusted for sociodemographic variables, behavioral, clinical conditions, and CVD-RF (see legend of **Table 2**).

^§^ In the interaction analyses, we included an interaction term (cognitive status * tooth loss).

^‖^ Cardiometabolic mortality combined diseases of heart, cerebrovascular diseases, and diabetes mellitus.

Boldface indicates statistical significance (*p* value < 0.05).

Abbreviations: HR, hazard ratio; CI, confidence interval; NHANES, National Health and Nutrition Examination Survey; CVD-RF, cardiovascular disease risk factors.
