# Supplementary material for: Combined association of cognitive impairment and poor oral health on mortality risk in older adults: Results from the NHANES with 15 years of follow‐up
Source: J Periodontol. 2021 Nov 12;93(6):888–900. doi: 10.1002/JPER.21-0292 (PMC9298999; doi:10.1002/JPER.21-0292)
Supplement: Supplementary file 10 — Supplemental Methods [file JPER-93-888-s003.docx]

**SUPPLEMENT METHODS**

*Estimating Healthy Eating Index (HEI)-2015 Scores*

The HEI-2015 score was designed based on the Dietary Guidelines for Americans to monitor changes in dietary patterns. The HEI-2015 contains 9 adequacy food components (total fruits, whole fruits, total vegetables, greens and beans, whole grains, dairy, total protein foods, seafood and plant proteins, and fatty acids) and 4 moderation food components (refined grains, sodium, added sugars, and saturated fats). We summed the scores for each component to calculate a total score (from 0 to 100). Each of the components is scored on a density basis out of 1,000 calories, with the exception of Fatty Acids, which is a ratio of unsaturated to saturated fatty acid. The simple HEI scoring algorithm method is applied to calculate scores using computed amounts of each component in the HEI. Details can be found on the NCI website: https://epi.grants.cancer.gov/hei/hei-scores-for-describing-dietary-intake.html. To use the simple HEI scoring method, first the ratio of the dietary constitutes to energy is constructed and scored according to the scoring standards. The component scores are summed to calculate the total score. The mean total score is the mean of the total scores across individuals. When more than one 24HR recall per person is available, the score is calculated by summing across all days per person before scoring.
